# An Improved Bio-Orientation Method Based on Direct Sunlight Compensation for Imaging Polarization Sensor

**DOI:** 10.3390/jimaging10040074

**Published:** 2024-03-24

**Authors:** Guangmin Li, Ya Zhang, Shiwei Fan, Fei Yu

**Affiliations:** School of Instrumentation Science and Engineering, Harbin Institute of Technology, Harbin 150001, China; 21b901030@stu.hit.edu.cn (G.L.); fanshiwei@hit.edu.cn (S.F.); yufei@hit.edu.cn (F.Y.)

**Keywords:** polarization orientation, imaging polarization sensor, polarization detection model, direct sunlight compensation

## Abstract

Direct sunlight in complex environmental conditions severely interferes with the light intensity response for imaging Polarization Sensor (PS), leading to a reduction in polarization orientation accuracy. Addressing this issue, this article analyzes the impact mechanism of direct sunlight on polarization sensor detection in a complex environment. The direct sunlight interference factor is introduced into the intensity response model of imaging polarization detection, enhancing the accuracy of the polarization detection model. Furthermore, a polarization state information analytical solution model based on direct sunlight compensation is constructed to improve the accuracy and real-time performance of the polarization state information solution. On this basis, an improved bio-orientation method based on direct sunlight compensation for imaging polarization sensor is proposed. The outdoor dynamic reorientation experiment platform is established to validate the effectiveness of the proposed method. Compared with the traditional methods, the experimental results demonstrate a 23% to 47% improvement in the polarization orientation accuracy under various solar zenith angles.

## 1. Introduction

Polarization is the high-dimensional information of light waves [1,2,3]. The imaging polarization sensor based on polarized light detection can provide high-precision, fully autonomous, and no-cumulative-error navigation information for intelligent unmanned platforms, providing reliable guarantees for the smooth completion of tasks. In clear weather, scattered particles are mainly composed of atmospheric molecules, with sizes much smaller than the wavelength of light. The atmospheric polarization pattern is mainly formed by single scattering. Therefore, a first-order Rayleigh scattering model can be used to describe the atmospheric scattering process under clear weather, where the direction of the electric vibration vector of the scattered light is perpendicular to the scattering surface. As sunlight traverses the Earth’s atmosphere, it establishes a stable polarization distribution pattern across the entire sky due to the scattering effects of atmospheric molecules and aerosol particles [4,5,6]. Biological studies have indicated that organisms such as mantis shrimp [7], octopuses [8], locust [9], and desert ants [10] can utilize their unique visual structures to perceive the polarization patterns of the entire sky, employing this information for self-orientation. Inspired by biomimetic navigation mechanisms, polarized navigation has demonstrated advanced performance in unmanned aerial vehicles [11,12], unmanned ground vehicles [13,14,15], and other navigation domains. With advantages such as no cumulative errors, resistance to electromagnetic interference, and robust concealment, it provides a novel solution for fully autonomous orientation in Global Navigation Satellite System (GNSS)-denied environments [16,17]. However, the current state of research on the interference error mechanism of polarization sensors is incomplete. Therefore, undertaking research on error models for PS in complex environments holds crucial significance for enhancing the accuracy of biomimetic polarized orientation.

Currently, polarization sensor error models can be categorized into point-source type based on photodiodes and imaging type based on Complementary Metal Oxide Semiconductor (CMOS) chips. For point-source PS, Lambrinos [18,19] utilized six sets of photodiodes to construct a point source PS, achieving heading measurements and conducting experiments on a mobile robot. Chu [20,21,22] built three pairs of polarization-opposing units using a polarization detector and logarithmic amplifier, calibrated the sensor using an integrating sphere, resulting in a polarization angle measurement error of less than 0.2°. Ma [23,24] proposed a polarization information calculation method based on the least squares method and calibrated the installation angle error and polarization angle error of the sensor. Dupeyroux [25] designed a UV polarization sensor, and conducted outdoor experiments in various weather and environmental conditions, achieving directional accuracy of less than 0.3° on clear days.

However, point-source PS can only obtain polarization information along a certain observation direction for a single measurement, making it susceptible to environmental occlusion and exhibiting poor robustness. Therefore, imaging polarization orientation methods have gained increasing attention. Sturzl [26,27] achieved the geometric calibration of a four-channel fisheye polarization camera and estimated the measurement covariance of the polarization channels. Chu [28,29] integrated double-layer nanowire polarizers with photodetectors using a one-time nanoimprint and metal deposition process, avoiding alignment errors associated with discrete polarizers. Fan [30] considered intensity response consistency errors and polarizer installation angle errors of PS. Ren [31] constructed an imaging PS model based on the extinction ratio error, further improving the accuracy of angle of polarization (AOP) detection. Li [32] proposed an on-site calibration method based on the Berry model, which improves the directional robustness in scenarios with severe multiple scattering. However, this method can mainly overcome the influence of the inaccurate polarization model caused by multiple scattering on the orientation results, and cannot adapt to the interference of direct sunlight on the detection results of the polarization sensor. Wan [33] further considered the distortion effects of the optical system in imaging PS and constructed the Mueller matrix of the optical system, resulting in a heading accuracy of 0.667°. However, the aforementioned methods did not account for the impact of direct sunlight interference in the environment on the polarization channels. Liu [34] developed a point-source PS detection model under direct sunlight interference, enhancing the sensor’s adaptability to different solar elevation angles. However, this method does not take into account the influence of optical path change caused by lens distortion on the imaging polarization model, and the polarization information estimation method based on least squares fitting is difficult to apply in real-time to imaging PS models. Additionally, some robust polarization orientation methods considering harsh scenarios did not account for direct sunlight interference [35,36,37], severely affecting heading measurement accuracy.

Motivated by these challenges, we propose a biomimetic orientation method for imaging polarization sensors based on direct sunlight compensation. Specifically, the main contributions of this paper are as follows:

(1) On the basis of the analyzing the interference of direct sunlight in complex environmental conditions on polarization sensor detection, the direct sunlight interference factor is introduced into the imaging polarization detection intensity response model, thereby enhancing the model’s accuracy.
(2) A polarization-state information analytical solution model is constructed by utilizing the redundant information of the four-channel polarization response intensity to formulate equations, which improves the accuracy and real-time performance of polarization state information resolution.
(3) A polarization heading measurement method based on direct sunlight compensation is proposed. And the simulation and outdoor dynamic reorientation experiment platform is established to verify the advancement of the proposed method.

The structure of this paper is organized as follows. Section 2 describes the existing problems with imaging PS, emphasizing that current PS models struggle to meet the requirements of high-precision autonomous navigation. Section 3 introduces the specific steps of the proposed improved bio-orientation method for imaging PS based on direct sunlight compensation. Section 4 presents simulations and outdoor experiments, discussing the experimental results. Finally, Section 5 summarizes the work and results of this paper.

## 2. Problem Description

For polarization navigation, the accuracy of the polarization sensor model directly affects that of heading measurement. To detect the polarization information of linearly polarized light generated by atmospheric scattering, multiple polarizers are usually required for a single polarization channel. The imaging polarization sensor usually has four polarization channels, and the installation angles of the polarizers are 0°, 45°, 90°, and 135°, respectively. Moreover, when light passes through a polarizer, its intensity will experience a certain attenuation, and the ratio of the attenuated intensity to the original intensity is defined as the extinction ratio. However, due to manufacturing process limitations, the installation angle of different polarizers can produce errors, and their extinction ratios are usually inconsistent. The resulting error parameters are defined as polarizer installation angle error and the extinction ratio inconsistency, respectively. In addition, after passing through the polarizer, light is received by the CMOS photosensitive chip and converted into a digital signal. The ratio of detection light intensity to incident light intensity is defined as the light intensity response coefficient. Similarly, the light intensity response coefficients of different pixel units in the CMOS photosensitive chip are different, and the resulting error parameter is defined as the light intensity response inconsistency. The traditional model of imaging polarization sensor can be expressed as follows:

(1) $$I_{out}^{k}=\frac{1}{2}\beta_{k}I_{in}\left[1+\eta_{k}d\cos\left(2\xi -2{\tilde{\alpha }}_{k}\right)\right],k=1,2,3,4$$

where $\beta_{k}$ is the parameter of the incident light intensity inconsistency of the *k*-th polarization channel, ${\tilde{\alpha }}_{k}=\alpha_{k}+\delta \alpha_{k}$ is the installation angle of the polarizer with error, and $\eta_{k}$ is the parameter of the extinction ratio inconsistency. ξ represents the AOP, and *d* represents the DOP. $I_{in}$ is the incident light intensity. $I_{out}^{k}$ is the output light intensity of $I_{in}$ after passing through the polarizer and CMOS.

**Figure 1:**
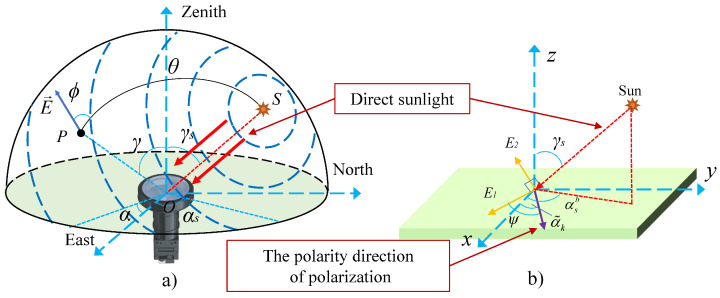
Polarization mechanism of direct sunlight passing the polarizer. (**a**) Direct sunlight incident on the imaging polarization sensor; (**b**) Direct sunlight incident on a single polarizer.

Although the traditional model improves the detection accuracy of scattered polarized light, it does not consider the impact of the coupling between direct sunlight and polarized light, seriously reducing the accuracy of polarization heading calculation. As shown in Figure 1a, when sunlight passes through the atmosphere, part of the light will be scattered by the atmosphere to form the full-sky polarization mode, while the other part is the direct sunlight that does not undergo polarization effect and is directly incident on the sensor. The detection process of direct sunlight does not meet the traditional polarization sensor model. According to the classical theory of wave optics, the direct sunlight can be described with the electric vector method. Thus, the intersection point between the incident light and the CMOS receiving plane can be used as the origin to establish a Cartesian coordinate system. Then $E_{1}$ represents the electrical component vector located in the sensor plane and perpendicular to the incident light, and $E_{2}$ represents the electrical component vector perpendicular to both the incident light and the $E_{1}$, as shown in Figure 1b. Therefore, model errors will be introduced during the calculation of polarization state, which seriously affects the accuracy of the polarization heading calculation. A more accurate sensor model needs to be proposed to decouple polarized light and direct sunlight, thereby improving the accuracy of heading measurement.

## 3. Method

This manuscript proposes a polarized orientation method based on direct sunlight compensation to address the aforementioned issues. The approach primarily encompasses three components: the construction of an improved imaging PS model with direct sunlight compensation, polarization state calculation, and polarization heading measurement.

### 3.1. Improved Imaging PS Model with Direct Sunlight Compensation

Figure 1 demonstrates the detection mechanism of direct sunlight inclined through a polarizer. Let OS represent the solar vector direction, and OP denote the observation vector direction. The electric field vector of direct sunlight can be decomposed into $E_{1}$ and $E_{2}$ components. $E_{1}$ lies on the surface of the polarizer, while $E_{2}$ is perpendicular to the plane formed by $E_{1}$ and OS. As $E_{1}$ passes through the polarizer, the transmitted light intensity can be expressed as follows:

(2) $$I_{out}^{E_{1}}=0.5I_{in}^{\mathrm{sun}}\cos^{2}\psi$$

$I_{in}^{\mathrm{sun}}$ represents incident light intensity of direct sunlight, $I_{out}^{E_{1}}$ represents the transmitted light intensity of $E_{1}$, and ψ represents the angle between $E_{1}$ and the polarity direction of the polarizer. Alternatively, following the fundamental transmission mechanism of polarized light [34], the intensity of the transmitted light through the polarizer for the electric field vector $E_{2}$ can be represented as follows:

(3) $$I_{out}^{E_{2}}=0.5I_{in}^{\mathrm{sun}}{\left(\cos\alpha -\frac{\sin\alpha }{\tan\beta }\right)}^{2}$$

where

(4) cosα=−sinψsinψsinγysinγycosβ=−sin2ψsin2γs−sin2ψsin2γs(2sinγysinγx)(2sinγysinγx)

$\gamma_{s}$ denotes the solar vector zenith angle, and ψ is the angle between $E_{1}$ and polarization direction. $\gamma_{x}$ represents the angle between the incident light and the x-axis, and $\gamma_{y}$ represents the angle between the incident light and the y-axis. As $\gamma_{s}$ tends towards 0, $\gamma_{x}$ and $\gamma_{y}$ are approaching 90°.

**Figure 2:**
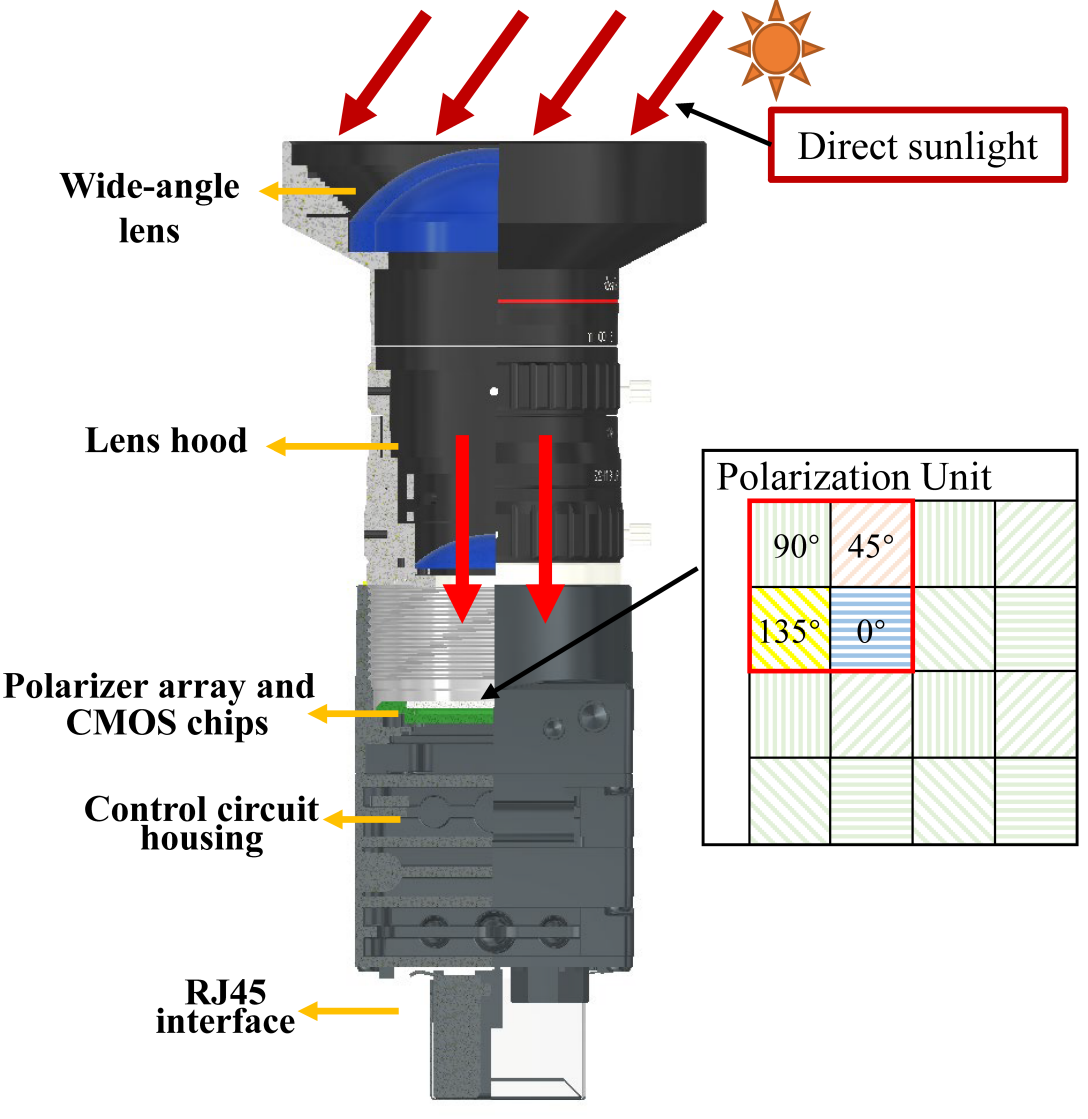
Schematic Diagram of Direct Sunlight Detection for Imaging PS.

Point-source PS is designed to directly probe the polarization information corresponding to the orientation of the CMOS chip. In contrast, imaging PS exhibits distinguishing characteristics, as delineated in Figure 2. When direct sunlight undergoes a wide-angle lens, alterations transpire in its light transmission path. Subsequently, after traversing the lens hood, the direct sunlight will incident upon the polarizer array and the CMOS chip in a direction approaching vertical. At this point, the solar zenith angle $\gamma_{s}$ of direct sunlight approximates zero. Consequently, the expression for outputting light intensity of $E_{2}$ is $I_{out}^{E_{2}}\approx 0.5I_{in}^{sun}\sin^{2}\psi$, enabling the derivation of the polarization response intensity expression for direct sunlight.

(5) $$\underset{\gamma_{s}\to 0}{I_{out}^{sun}}\;=I_{out}^{E_{1}}+I_{out}^{E_{2}}\approx 0.5I_{in}^{\mathrm{sun}}\left(\cos^{2}\psi +\sin^{2}\psi \right)=0.5I_{in}^{\mathrm{sun}}$$

However, in the actual measurement process, $I_{in}^{sun}$ is usually a part of the total light intensity and varies with the azimuth angle  ψ. Therefore, by analogy with Equation (2), $I_{out}^{sun}$ is redefined:

(6) $$I_{out}^{sun}=0.5I_{in}k_{m}\cos^{2}\psi =I_{in}k_{m}P$$

*P* represents the polarization response coefficient of direct sunlight, which is related to ψ. Then, the cosine duplication formula is used to expand Equation (1):

(7) $$I_{out}^{k}=\beta_{k}\left[I_{in}\eta_{k}d\cos^{2}\left(\xi -{\tilde{\alpha }}_{k}\right)+0.5I_{in}\left(1-\eta_{k}d\right)\right]$$

The above formula indicates that the intensity of the non-completely polarized light detected by the sensor is equal to the sum of the polarized light intensity and the non-polarized light intensity. The unpolarized light intensity represented by the second term on the right side of the equation is composed of atmospheric scattering polarized light and direct sunlight. Define $k_{m}$ as the intensity of direct sunlight weight in the total unpolarized light intensity, then the above formula can be rewritten as follows:

(8) $$I_{out}^{k}=\beta_{k}\left[I_{in}\eta_{k}d\cos^{2}\left(\xi -{\tilde{\alpha }}_{k}\right)+0.5I_{in}\left(1-\eta_{k}d\right)\left(1-k_{m}\right)+P_{k}I_{in}\left(1-\eta_{k}d\right)k_{m}\right]$$

The cosine squared term on the right side of the equation is transformed into a double angle form and combined with the second and third terms to obtain the following:

(9) $$I_{out}^{k}=\frac{1}{2}\beta_{k}\left[1+I_{in}\eta_{k}d\cos\left(2\xi -2{\tilde{\alpha }}_{k}\right)\right]+0.5\beta_{k}I_{in}\left(1-\eta_{k}d\right)k_{m}\left(P_{k}-0.5\right)$$

The imaging polarization detection model of a single channel for the four-channel polarization unit can be obtained, which considers direct sunlight interference.

The error-free installation angles of the polarizer corresponding to ${\tilde{\alpha }}_{k}$ are 0°, 45°, 90°, and 135°; $P_{k}$ represents the polarization response coefficient of direct sunlight in the *k*-th channel, which can be calculated by the angle $\psi_{k}$ between the electric field vector $E_{1}$ and the polarizer:

(10) $$P_{k}=0.5\cos^{2}\psi_{k}$$

$\psi_{k}$ is the angle between the azimuth angle of $E_{1}$ and the installation direction of the polarizer. The azimuth angle of $E_{1}$ differs by π/2 from the azimuth angle $\alpha_{s}^{b}$ of the solar vector under the carrier system. In navigation systems, when the camera installation error angle is calibrated, it is usually considered that the carrier coordinate system coincides with the camera coordinate system. Therefore, the carrier coordinate system in this article is equivalent to the camera coordinate system, which can be established on the sensor plane with the intersection point of the optical axis and the image plane as the origin, as shown in Figure 1b. At this point, $\alpha_{s}^{b}$ is the angle between the xoy-plane projection of the sun vector in the carrier coordinate system and the *x*-axis. Therefore, by defining the counterclockwise direction as positive, the expression for $\psi_{k}$ can be obtained as follows:

(11) $$\psi_{k}=\left(\alpha_{s}^{b}-\pi /2\right)-{\tilde{\alpha }}_{k}$$

In summary, after organizing Equation (9), the final PS detection model under direct sunlight interference can be obtained:

(12) $$\left\{\begin{array}{c} I_{out}^{k}=\frac{1}{2}\beta_{k}I_{in}\left[1+\eta_{k}d\cos\left(2\xi -2{\tilde{\alpha }}_{k}\right)\right]+I_{out_{k}}^{sun}\\ I_{out_{k}}^{sun}=0.5\beta_{k}I_{in}\left(1-\eta_{k}d\right)k_{m}\left(P_{k}-0.5\right) \end{array}\right.$$

Compared with the traditional model, as shown in Equation (1), the improved imaging PS detection model additionally considers the impact of direct sunlight. $I_{out_{k}}^{sun}$ represents the intensity response value of direct sunlight transmitted through the polarizer to the CMOS chip. Therefore, the environmental adaptability of the traditional model could be improved. In addition, when the direct sunlight weight $k_{m}$ is zero, the improved PS model as shown in Equation (12) degenerates to the traditional model. It is noteworthy that the solar azimuth $\alpha_{s}^{b}$ in the carrier system in Equation (11) can be obtained through the solar azimuth in the navigation coordinate system and the approximate heading of the carrier:

(13) $$\alpha_{s}^{b}=\alpha_{s}^{n}-heading_{n}$$

$\alpha_{s}^{n}$ is obtained by incorporating the geographical position and time of the carrier into the solar ephemeris; $heading_{n}$ can be provided by other navigation systems, such as an inertial navigation system.

### 3.2. Analytical Solution of Polarization State Based on Improved Imaging PS Model

After establishing the detection model of the imaging polarization sensor, it is necessary to further combine response light intensity of the measured polarization channels to solve the polarization state. Therefore, a mapping model between the detection value $I_{m}^{k}$ and the Stokes vector s needs to be established. The traditional polarization state calculation method based on Mode1 is as follows:

(14) $$s=\left[\begin{array}{c} s_{0}\\ s_{1}\\ s_{2} \end{array}\right]=\left[\begin{array}{c} I_{in}\\ I_{in}d\cos2\xi \\ I_{in}d\sin2\xi \end{array}\right]=\left[\begin{array}{c} I_{m}^{1}+I_{m}^{3}\\ I_{m}^{3}-I_{m}^{1}\\ I_{m}^{4}-I_{m}^{2} \end{array}\right]$$

$I_{m}^{k}$ is the measured value of polarization response light intensity for a single channel. From this, the rough angle of polarization ξ and degree of polarization *d* can be calculated:

(15) $$\xi =\arctan\left(s_{2},s_{1}\right)/2,d=\sqrt{{s_{1}}^{2}+{s_{2}}^{2}}/s_{0}$$

Although this method does not consider the influence of direct sunlight, we can use the calculated degree of polarization *d* and incident light intensity $I_{in}$ as prior information for the proposed algorithm. For the PS model under direct sunlight interference, Equation (8) can be expanded to obtain the following:

(16) $${\hat{I}}_{out}^{k}=I_{in}+\eta_{k}\cos2{\tilde{\alpha }}_{k}\cdot I_{in}d\cos2\xi +\eta_{k}\sin2{\tilde{\alpha }}_{k}\cdot I_{in}d\sin2\xi +{\hat{P}}_{k}\cdot I_{in}k_{m}$$

where ${\hat{I}}_{out}^{k}=I_{out}^{k}/\beta_{k}$ and ${\hat{P}}_{k}=0.5\left(1-\eta_{k}d\right)\left(P_{k}-0.5\right)$. The solution process of the polarization state is to determine the parameter set $\left\{\beta_{k},\eta_{k},{\tilde{\alpha }}_{k},k_{m}|k=1,2,3,4\right\}$ and make it satisfy the following polarization channel light intensity response objective function:

(17) $$\min_{\left\{\beta_{k},\eta_{k},{\tilde{\alpha }}_{k},k_{m}\right\}}\;\sum_{k=1}^{4}{∥I_{m}^{k}-I_{out}^{k}∥}^{2}$$

$\left\{\beta_{k},\eta_{k},{\tilde{\alpha }}_{k}\right\}$ can be obtained through prior calibration, while $k_{m}$ is mainly determined by the interference of direct sunlight, which is closely related to the carrier environment and needs to be solved in real time. Therefore, this article deduces the solution process of $k_{m}$ and the polarization state in detail. We extend the Stokes vector s and rewrite Equation (16) in linear matrix form.

(18) $$s={\left[\begin{array}{cccc} s_{1} & s_{2} & s_{3} & s_{4} \end{array}\right]}^{T}={\left[\begin{array}{cccc} I_{in} & I_{in}d\cos2\xi & I_{in}d\sin2\xi & I_{in}k_{m} \end{array}\right]}^{T}$$

(19) $$F=\left[\begin{array}{c} {\hat{I}}_{out}^{1}\\ {\hat{I}}_{out}^{2}\\ {\hat{I}}_{out}^{3}\\ {\hat{I}}_{out}^{4} \end{array}\right],D=\left[\begin{array}{cccc} 1 & \eta_{1}\cos2{\tilde{\alpha }}_{1} & \eta_{1}\sin2{\tilde{\alpha }}_{1} & {\hat{P}}_{1}\\ 1 & \eta_{2}\cos2{\tilde{\alpha }}_{2} & \eta_{2}\sin2{\tilde{\alpha }}_{2} & {\hat{P}}_{2}\\ 1 & \eta_{3}\cos2{\tilde{\alpha }}_{3} & \eta_{3}\sin2{\tilde{\alpha }}_{3} & {\hat{P}}_{3}\\ 1 & \eta_{4}\cos2{\tilde{\alpha }}_{4} & \eta_{4}\sin2{\tilde{\alpha }}_{4} & {\hat{P}}_{4} \end{array}\right]$$

Equations (18) and (19) satisfy Ds=F. Therefore, the least squares method can be used to solve Equation (17) to obtain the optimal Stokes vector as

(20) $$\hat{s}={\left(D^{T}D\right)}^{-1}\cdot D^{T}F$$

We can use Equation (20) to solve the Stokes vector of a single polarization channel based on direct sunlight interference. However, for imaging PS with millions of polarization channels, the matrix inversion operation in Equation (20) will consume a lot of time. Therefore, it is necessary to derive an analytical solution method for $\hat{s}$. We ignore the influence of the installation angle error $\delta \alpha_{k}$ of the polarizer and the extinction ratio error $\eta_{k}$, and incorporate installation angle ${\tilde{\alpha }}_{k}$ into Equation (16) to obtain the equation set:

(21) $$\left\{\begin{array}{c} {\hat{I}}_{out}^{1}=I_{in}+I_{in}d\cos2\xi +{\hat{P}}_{1}I_{in}k_{m}\\ {\hat{I}}_{out}^{2}=I_{in}+I_{in}d\sin2\xi +{\hat{P}}_{2}I_{in}k_{m}\\ {\hat{I}}_{out}^{3}=I_{in}-I_{in}d\cos2\xi +{\hat{P}}_{3}I_{in}k_{m}\\ {\hat{I}}_{out}^{4}=I_{in}-I_{in}d\sin2\xi +{\hat{P}}_{4}I_{in}k_{m} \end{array}\right.$$

Thus, the weight $k_{m}$ of direct sunlight in non-polarized light can be calculated:

(22) $$k_{m}=k_{c}\frac{\left({\hat{I}}_{out}^{1}+{\hat{I}}_{out}^{2}+{\hat{I}}_{out}^{3}+{\hat{I}}_{out}^{4}\right)-4I_{in}}{I_{in}\cdot \left({\hat{P}}_{1}+{\hat{P}}_{2}+{\hat{P}}_{3}+{\hat{P}}_{4}\right)}$$

$k_{c}$ is a constant, and its physical meaning is the compensating for the theoretical error of the analytical method compared to the least squares method. In this paper, its empirical value is taken as 20. Then we can obtain the corresponding light intensity value of the polarization channel after direct sunlight compensation:

(23) $${\hat{I}}_{m}^{k}=I_{m}^{k}-{\hat{P}}_{k}I_{in}k_{m},k=1,2,3,4$$

Finally, we can obtain the Stokes vector after direct sunlight compensation, as shown in Equation (24).

(24) $$\hat{s}={\left[\begin{array}{ccc} {\hat{s}}_{1} & {\hat{s}}_{2} & {\hat{s}}_{3} \end{array}\right]}^{T}={\left[\begin{array}{ccc} {\hat{I}}_{m}^{1}+{\hat{I}}_{m}^{3} & {\hat{I}}_{m}^{3}-{\hat{I}}_{m}^{1} & {\hat{I}}_{m}^{4}-{\hat{I}}_{m}^{2} \end{array}\right]}^{T}$$

According to Equation (15), the AOP measurement value $\hat{\xi }=\arctan\left({\hat{s}}_{2},{\hat{s}}_{1}\right)$ after direct sunlight compensation can be obtained.

### 3.3. Heading Measurement

**Figure 3:**
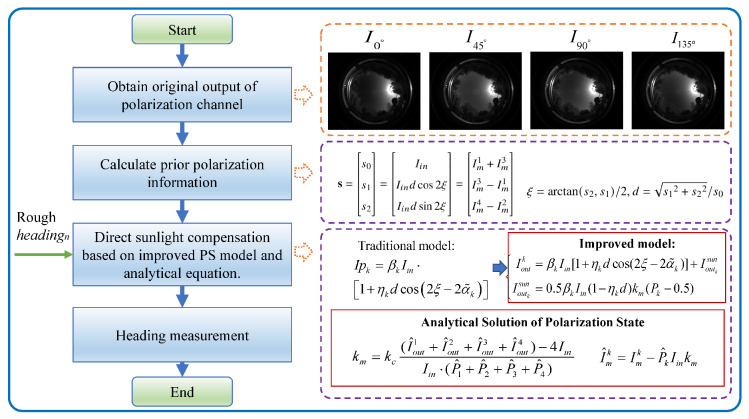
Flow chart of polarization orientation method based on direct sunlight compensation.

After obtaining the AOP value $\hat{\xi }$ compensated for direct sunlight in the sensor coordinate system, it is necessary to further utilize polarimetric information to calculate the carrier’s heading, as shown in Figure 3. On the basis of obtaining raw polarization data and prior polarization information, the improved polarization detection model under direct sunlight interference and the analytical equation of the polarization state is constructed. Therefore, we can acquire more accurate polarization state information of incident light, thereby improving heading measurement accuracy. The specific steps for heading measurement are as follows.

**Figure 4:**
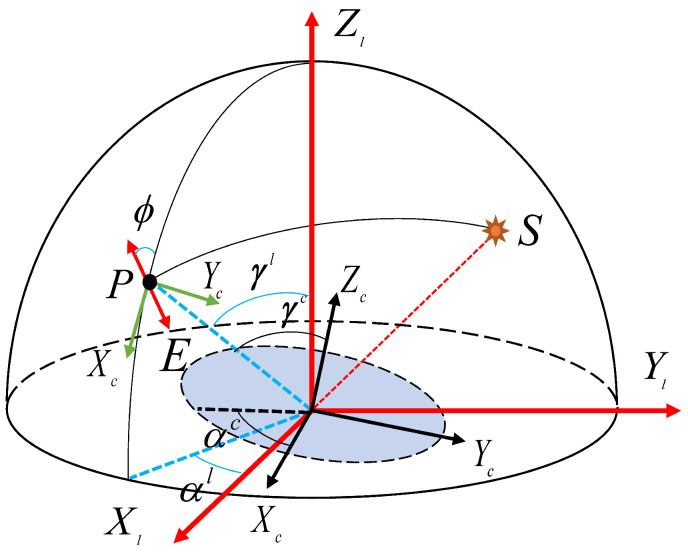
The representation for *E*-vector of different reference coordinate systems in tilted state.

The Rayleigh model is employed to characterize the polarization pattern throughout the entire sky, where the polarization *E*-vector direction is perpendicular to the scattering surface. Additionally, we consider the influence of the carrier tilt on heading measurements. The representation for *E*-vector of different reference coordinate systems in tilted state is shown in Figure 4. *S* represents the sun position, and *P* represents the observation position.

Assuming that the carrier coordinate system coincides with the sensor coordinate system, where the *c*-frame represents the sensor coordinate system, the *l*-frame represents the horizontally referenced coordinate system after tilt correction, and the n-frame represents the “East-North-Up” geographical coordinate system. Figure 4 shows the AOP measurement process in the inclined state from the local meridian through the observation celestial sphere. $\gamma^{l}$ and $\alpha^{l}$ is the zenith angle and azimuth of the observation point under the horizontal reference frame. The expression in *l*-frame of $\vec{OP^{c}}$ is

(25) $$\vec{OP^{l}}={\left(R_{l}^{c}\right)}^{T}\vec{OP^{c}}$$

where $\vec{OP^{c}}$ is the observation vector under the carrier system, and it can be obtained through camera calibration of internal parameters and distortion parameters. The transformation matrix from *l*-frame to *c*-frame is

(26) $$R_{l}^{c}=\left[\begin{array}{ccc} 1 & 0 & 0\\ 0 & \cos ro & \sin ro\\ 0 & -\sin ro & \cos ro \end{array}\right]\times \left[\begin{array}{ccc} \cos pi & 0 & -\sin pi\\ 0 & 1 & 0\\ \sin pi & 0 & \cos pi \end{array}\right]$$

ro and pi represent the roll and pitch angle of the camera. Thus, the zenith angle and azimuth in *l*-frame after inclination compensation can be obtained.

(27) $$\gamma^{l}=\arccos\left(\vec{OP^{l}}\left(3\right)\right),\alpha^{l}=\arctan\left(\vec{OP^{l}}\left(2\right)/\vec{OP^{l}}\left(1\right)\right)$$

According to [38], ϕ represents the angle between the E vector and the observed meridian plane. The relationship between ϕ and $\hat{\xi }$ is represented as

(28) $$\tan\phi =\frac{\cos\gamma^{l}\left(\tan\psi -\tan\alpha^{l}\right)}{\tan\psi \tan\alpha^{l}+1}=\cos\gamma^{l}\tan\left(\psi -\alpha^{l}\right)$$

Furthermore, in order to calculate the solar vector e using the Rayleigh scattering model, it is necessary to calculate the expression of the polarization vector in *n*-frame:

(29) $$e^{l}=\vec{PE^{l}}=\cos\phi \vec{PX^{l}}+\sin\phi \vec{PY^{l}}$$

where ${\vec{PX}}_{w}$ and ${\vec{PY}}_{w}$ are the coordinate base vectors of the *y*-axis and *x*-axis in *w*-frame, which can be expressed as

(30) $$\left\{\begin{array}{c} \vec{PX^{l}}={\left(\begin{array}{ccc} \cos\alpha^{l}\cos\gamma^{l} & \sin\alpha^{l}\cos\gamma^{l} & -\sin\gamma^{l} \end{array}\right)}^{T}\\ \vec{PY^{l}}={\left(\begin{array}{ccc} -\sin\alpha^{l} & \cos\alpha^{l} & 0 \end{array}\right)}^{T} \end{array}\right.$$

By incorporating Equation (30) into Equation (29), the polarization vector $e^{l}$ compensated for the inclination error in *l*-frame. Further, according to the single Rayleigh scattering model, the vector *E* of the scattered light is perpendicular to the scattering plane, and in the same way, the vector *E* is perpendicular to the solar vector $\vec{OS^{l}}$, which is denoted as ${\left(e^{l}\right)}^{T}\vec{OS^{l}}=e^{l}{\vec{OS^{l}}}^{T}$. Therefore, define $E=[e_{1}^{l}\ldots e_{N}^{l}]$, in which *N* represents the number of valid pixels, so that $E^{T}s^{l}=0$. Solving the solar vector in *l*-frame can be expressed as an optimization problem as follows

(31) $$\min_{s}\left({\left(s^{l}\right)}^{T}EE^{T}s^{l}\right),\;s.t.\;{\left(s^{l}\right)}^{T}s^{l}=1$$

The characteristic vector λ corresponding to the minimum eigenvalue of the matrix $EE^{T}$ is the best estimate of the solar vector, which can be obtained by singular value decomposition (SVD). The solar meridian direction in *l*-frame is $\alpha_{sun}^{l}=\arctan\left(\lambda \;\left(2\right)/\lambda \;\left(1\right)\right)$. λ(1) and λ(2) are the first and second elements, respectively.

According to the celestial ephemeris, the sun’s zenith angle $\gamma_{S}$ and azimuth angle $\alpha_{S}$ in the navigation coordinate system can be determined from local time and position. The heading can be calculated from the difference of the azimuth angle in *n*-frame and the measured values $\alpha_{sun}^{l}$ in *l*-frame:

(32) $$heading=\alpha_{S}-\alpha_{sun}^{l}\;\mathrm{or}\;heading=\alpha_{S}-\alpha_{sun}^{l}+\pi$$

There is 180° ambiguity in the solution of the heading angle, which can be determined by the integrated navigation system.

In summary, the specific steps for processing a single frame image and outputting the heading using the anti-interference orientation method based on direct sunlight compensation by imaging polarization sensor are shown in Algorithm 1.    
**Algorithm 1** Polarization orientation method based on direct sunlight compensation. **Data**: Measured polarization intensity ${\left\{I_{m}^{k,i}\right\}}_{i=1}^{N}\;$,    carrier attitude pi and ro, and  $heading_{n}$ **Result**: Polarized heading heading after direct sunlight compensation 1: Initialize the prior value ${\left\{d_{i}\right\}}_{i=1}^{N}$,${\left\{I_{in}^{i}\right\}}_{i=1}^{N}$; 2: ${\left\{{\hat{I}}_{out}^{k,i}\right\}}_{i=1}^{N}\leftarrow I_{out}^{k}/\beta_{k}$,$\;\{{\hat{P}}_{k}^{i}{\}}_{i=1_{k}}^{N}\leftarrow 0.5\left(1-\eta_{k}d\right)\left(P_{k}-0.5\right)$; 3: Calculate the direct sunlight weights ${\left\{k_{m}^{i}\right\}}_{i=1}^{N}$ from Equation (22); 4: ${\left\{{\hat{I}}_{m}^{k,i}\right\}}_{i=1}^{N}\leftarrow I_{m}^{k}-{\hat{P}}_{k}I_{in}k_{m}$, and calculate ${\left\{{\hat{s}}_{i}\right\}}_{i=1}^{N}$ and ${\left\{{\hat{\xi }}_{i}\right\}}_{i=1}^{N}$ from Equations (23) and (24); 5: $E=\left[e_{1}^{l}...e_{N}^{l}\right]\leftarrow \cos\phi \vec{PX^{l}}+\sin\phi \vec{PY^{l}}$; 6: $\alpha_{sun}^{l}\leftarrow \min_{s}\left({\left(s^{l}\right)}^{T}EE^{T}s^{l}\right),\;s.t.\;{\left(s^{l}\right)}^{T}s^{l}=1$; 7: Obtain the polarization orientation heading from Equations (31) and (32); 8: return heading

## 4. Experimental Result and Discussion

In this section, the simulation and outdoor experiment are carried out to verify the effectiveness of the polarization orientation method based on direct sunlight compensation proposed in this paper. We use the final heading measurement accuracy as the evaluation indicator.

### 4.1. Simulation

In this part, we simulate the image PS output in the direct sunlight interference scenario, and carry out polarization orientation based on different sensor models to verify the advantage of the proposed method. The specific simulation parameter settings are shown in Table 1. $\beta_{k}$, $\eta_{k}$, and ${\tilde{\alpha }}_{k}$ are set according to prior calibration. Direct sunlight weight parameter $k_{m}$ is set to 0.05. The camera’s internal parameters $\left[k_{c,x},k_{c,y},f_{c,x},f_{c,y}\right]$ and fisheye lens distortion parameters $\left[D_{0},D_{1},D_{2},D_{3}\right]$ are also set based on prior calibration values. Finally, we set the sun vector, geographic longitude and latitude where the carrier is located.

**Figure 5:**
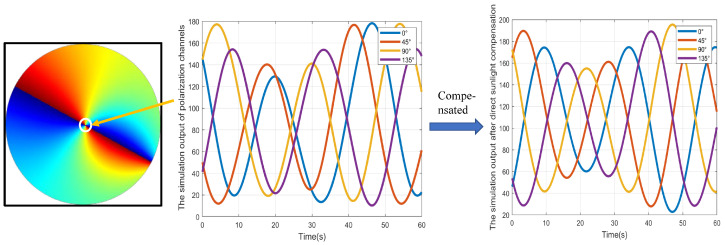
Simulation results of polarization channel under direct sunlight interference.

By setting the rotation rate of the PS to 720° in 100 s, the output of the light intensity under direct sunlight interference for multiple polarization channels can be obtained. The output of a single polarization channel in the central region is shown in Figure 5. The left image represents a simulated AOP image. The middle image represents the intensity of light output by the four polarization channels in the central region, which is affected by direct sunlight interference. The image on the right represents the polarization channel output after direct sunlight compensation. It can be seen that the amplitude and phase for imaging PS under different polarization directions are obviously disturbed. After direct sunlight compensation, although there is still a slight difference in amplitude, the phase and period of the compensated polarization channel show significant consistency. This is because the optical center of the simulated camera is not consistent with the pixel center, and the position of the observation vector cannot completely coincide with the zenith direction during rotation, resulting in differences in the polarization information source of the observation direction itself. The progressiveness of the method will be further verified through heading measurement experiments.

**Figure 6:**
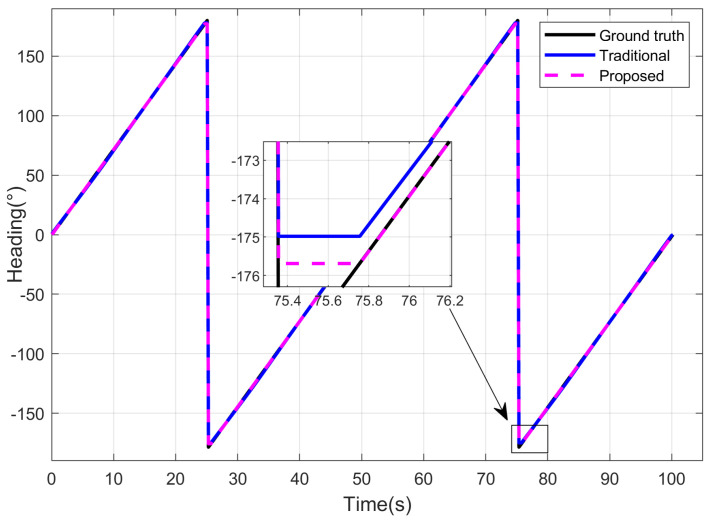
Heading results of different methods for simulation experiment.

Then we use polarization orientation methods based on different PS models for polarization heading calculation. The traditional method does not consider the impact of direct sunlight [32,33,39], while the proposed method adopts the improved polarization detection model for orientation. In the proposed method, a normal distribution random noise with a mean of 0° and a variance of 0.5° was applied to the heading reference value, serving as the prior heading ($heading_{n}$). In the simulation experiment, the heading measurement results of different methods are depicted in Figure 6. It can be observed that the proposed method, incorporating direct sunlight compensation, effectively suppresses the impact of direct sunlight on the detected intensity for the polarization channel. Compared to the traditional method, the directional measurement results of the proposed model more closely approximate the true heading.

**Figure 7:**
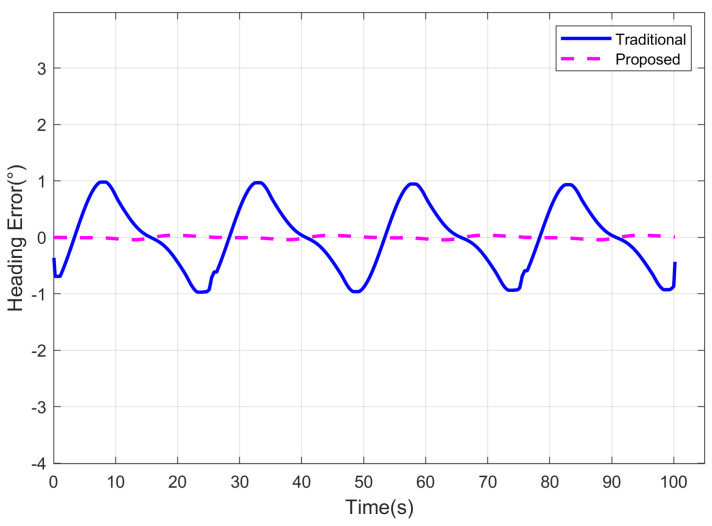
Heading errors of different methods for simulation experiment.

The heading errors of different methods are illustrated in Figure 7. In the scenario with direct sunlight interference, the traditional method exhibits significant fluctuation errors in heading measurements due to the absence of a corresponding error suppression mechanism. In contrast, the proposed method addresses this issue by constructing a sensor detection model under direct sunlight interference and deriving a corresponding polarization channel intensity error compensation algorithm. This enhancement improves the accuracy of the AOP measurement, consequently reducing the heading errors for imaging PS. In the simulation experiments, the heading Root Mean Square Error (RMSE) for the traditional method is 0.5928°, while the proposed method achieves a significantly lower RMSE of 0.0240°. Subsequent sections will further validate the sophistication of the proposed algorithm through outdoor experiments.

### 4.2. Outdoor Experiment

**Figure 8:**
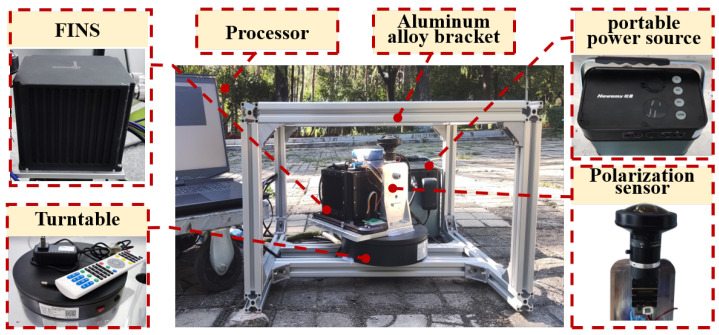
Diagram of the equipment for the outdoor experiment.

In this section, outdoor experiments were conducted to validate the proposed methods’s heading measurement performance under actual direct sunlight interference. The equipment used in the outdoor experiment is depicted in Figure 8. The imaging PS system consists of a polarized camera and a fisheye lens. The heading accuracy of the Fiber Optic Inertial Navigation System (FINS) is up to 0.02°/secL, where secL=1/cosL, and *L* represents the latitude of the location. And it is sufficient to be used as the reference for polarization orientation. The specific parameters of the equipment are detailed in Table 2. The experiment was conducted near Harbin (longitude 126.7264°, latitude 45.6234°, altitude 148.74 m) on 5 September 2023.

During the experiment, the initial alignment of FINS was conducted first, followed by the controlled rotation of the turntable to capture full-sky polarization images at different azimuths during various time intervals throughout the day.

**Figure 9:**
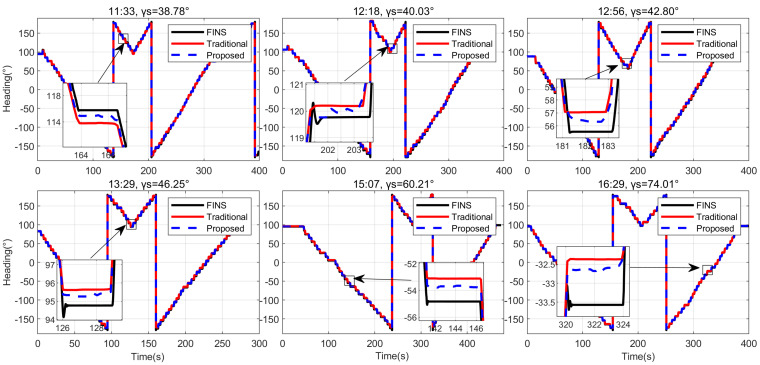
Heading results of different methods for outdoor experiment with different solar zenith angles.

**Figure 10:**
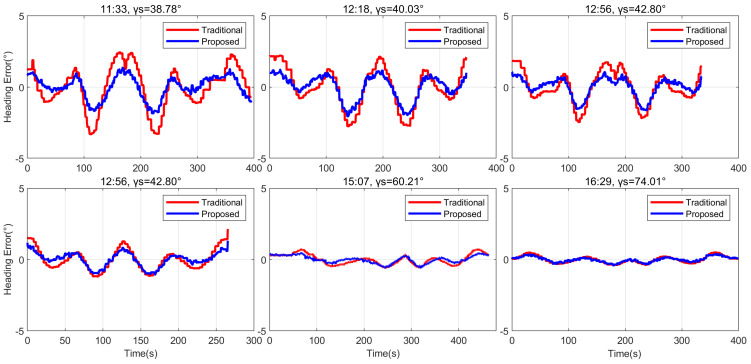
Heading errors of different methods for outdoor experiment with different solar zenith angles.

The heading results of various methods in outdoor experiments are depicted in Figure 9. $\gamma_{s}$ represents the zenith angle of the sun. Both the traditional method and the proposed method exhibit proficient capabilities in computing the carrier’s heading information. However, due to the consideration of the influence of direct sunlight, the proposed method demonstrates a closer approximation to the reference heading compared to traditional models even in actual datasets. Subsequently, a quantitative analysis of the heading results, as illustrated in Figure 10, reveals that the heading errors of different methods closely align with simulation results, exhibiting a fluctuation amplitude dependent on the orientation of the carrier.

Notably, the proposed method excels in suppressing the impact of direct sunlight on the polarized channel by incorporating compensation for direct sunlight effects, consequently achieving higher heading accuracy across different solar elevation angles. Under conditions of high solar zenith, the proposed model’s heading measurement error is observed to be less than 0.1843°. The RMSE of heading measurement for different methods, presented in Table 3, indicates that the heading errors of the proposed method are reduced by 34.52% on average, compared to the traditional method under varying solar zenith. This underscores the advancement and expansive application prospects of the proposed methodology. Additionally, as the solar zenith angle decreases, the proportional reduction in heading errors by the proposed method also diminishes, as shown in Figure 10. This is attributed to the higher proportion of direct sunlight in non-polarized light under smaller solar zenith angles, emphasizing the pronounced effectiveness of sunlight error suppression and further highlighting the high adaptability of our proposed method to diverse environmental conditions.

In addition, the proposed method has certain advantages over sun sensors in occluded scenes. When there is interference from leaves or clouds, even if the sun is blocked, there will still be strong direct sunlight in the obstructed edge area. In this case, the sun sensor will find it difficult to calculate the direction of the sun due to the obstruction of the sun within the field of view. The proposed method based on imaging polarization sensors can calculate the sun vector without directly observing the sun, and can improve the orientation accuracy through the direct sunlight compensation method, which has a broader application space.

## 5. Conclusions

To address the requirements for the high-accuracy and fully autonomous orientation of unmanned platforms in GNSS-denied environments, this paper focuses on the significant interference of direct sunlight in the light-intensity response process of imaging PS in complex environmental conditions, leading to a reduction in polarization orientation accuracy. Direct sunlight interference factors are incorporated into the intensity response model, enhancing the accuracy of the polarization detection model. Moreover, the polarization state information analytical solution model with direct sunlight compensation is constructed to improve the accuracy and real-time performance of polarization state information resolution. In practical outdoor experiments, the heading error is reduced by 23% to 47% under various solar zenith angles compared to the traditional method, confirming the advancement and broad application prospects of the proposed method. Future research will delve into polarization orientation methods in complex scenarios such as underwater and urban canyons to enhance the robustness of polarization navigation.

## Figures and Tables

**Table 1:** Parameters for simulation settings.

Parameters	Value
$I_{in}$	220Lx
$\beta_{k}$	[1.0269, 1.0, 1.038, 1.005]
$\eta_{k}$	[1, 1, 1, 1]
${\tilde{\alpha }}_{k}$	[0°, 45°, 90°, 135°]
$k_{m}$	0.05
$\left[k_{c,x},k_{c,y}\right]$	[622.49, 515.06]
$\left[f_{c,x},f_{c,y}\right]$	[259.10, 259.20]
$\left[D_{0},D_{1},D_{2},D_{3}\right]$	[0.029274, −0.013300, 0.009758, −0.004919]
Longitude	126.7264°
Latitude	45.6234°
$\alpha_{s}^{n}$	247.11°
$\gamma_{s}^{n}$	60.21°

**Table 2:** Instrument specifications.

Device Name	Device Model	Parameters
Lucid polarization camera	PHXET050S-P	Resolution: 2048 × 2448, Frame rate: 22 FPS
Fujinon Fisheye Lens	FE185C057HA-1	Focal length: 1.8 mm, view: 185.0° × 185.0°
FINS	—	Bias: 0.02 mg (1σ) (Accelerometer); Bias: 0.02°/*h* (1σ), ARW: 0.002°/$\sqrt{h}$ (Gyro).

**Table 3:** Standard deviation of heading error of different models for outdoor experiment.

	1	2	3	4	5	6	Average
Time	11:33	12:18	12:56	13:29	15:07	16:29	-
Traditional	1.4931	1.3902	1.0988	0.7302	0.3546	0.2418	0.8848
Proposed	0.7905	0.8700	0.6574	0.4983	0.2590	0.1843	0.5433
Ratio	47.06%	37.42%	40.17%	31.76%	26.96%	23.78%	34.52%

## Data Availability

The datasets used and/or analyzed during the current study are available from the corresponding author on reasonable request.
